# Cellular microparticles and pathophysiology of traumatic brain injury

**DOI:** 10.1007/s13238-017-0414-6

**Published:** 2017-05-02

**Authors:** Zilong Zhao, Yuan Zhou, Ye Tian, Min Li, Jing-fei Dong, Jianning Zhang

**Affiliations:** 1 Department of Neurosurgery, Tianjin Institute of Neurology, Tianjin Medical University General Hospital, Tianjin, 300052 China; 2 BloodWorks Northwest Research Institute, Seattle, WA 98102 USA; 3 Institute of Pathology, School of Basic Medical Sciences, Lanzhou University, Lanzhou, 730000 China; 4 Division of Hematology, Department of Medicine, School of Medicine, University of Washington, Seattle, WA 98195 USA

**Keywords:** traumatic brain injury, cellular microparticles, coagulopathy, inflammation

## Abstract

Traumatic brain injury (TBI) is a leading cause of death and disability worldwide. The finding that cellular microparticles (MPs) generated by injured cells profoundly impact on pathological courses of TBI has paved the way for new diagnostic and therapeutic strategies. MPs are subcellular fragments or organelles that serve as carriers of lipids, adhesive receptors, cytokines, nucleic acids, and tissue-degrading enzymes that are unique to the parental cells. Their sub-micron sizes allow MPs to travel to areas that parental cells are unable to reach to exercise diverse biological functions. In this review, we summarize recent developments in identifying a casual role of MPs in the pathologies of TBI and suggest that MPs serve as a new class of therapeutic targets for the prevention and treatment of TBI and associated systemic complications.

## Introduction

Traumatic brain injury (TBI) is a leading cause of death and disability among adolescent males and young adults. Approximately 3.5 million TBI cases are reported each year in emergency rooms throughout the United States, resulting in approximately 50,000 annual deaths (Cuthbert et al., 2015; He et al., 2005). TBI undergoes two distinct, but partially overlapping phases of primary and secondary injures. The primary injury occurs at the moment of trauma as a result of mechanical forces that disrupt the structural integrity of the brain. It rapidly evolves into secondary biochemical and cellular changes (Ghajar, 2000). The interplay between the brain and other organs propagates oxidative, hemostatic, ischemic, and inflammatory injuries secondary to TBI. Cellular microparticles produced by injured tissues have been increasingly recognized as a key mediator for the interplay, promoting a transition from primary injury to secondary injury (Maas et al., 2010; Stoica and Faden, 2010). This review focuses on systemic impacts of cellular microparticles released from traumatized brains, with specific emphasis on TBI-associated coagulopathy (TBI-AC) and inflammation.

## Cellular microparticles

MPs are a class of subcellular vesicles that are composed of shed membrane fragments and intracellular organelles and nuclear components. They are produced from cells undergoing active microvesiculation (Siljander et al., 2001; Heemskerk et al., 1997; Alkhamis et al., 1990; Owens and Mackman, 2011) or apoptosis (Shcherbina and Remold-O’Donnell, 1999; Dale and Friese, 2006; Brown et al., 2000). The former is triggered by the activation of the cysteine protease calpain, which disrupts the membrane-cytoskeleton association (Fox et al., 1991; Fox et al., 1990; Saatman et al., 2010; Zetterberg et al., 2013). This is a self-propelling mechanism whereby increasing intracellular Ca^2+^ induces glutamate release to activate the N-methyl-D-aspartate (NMDA) receptor, leading to further increases in cytosolic and mitochondrial Ca^2+^ levels (Cheng et al., 2012). The current definition of MPs is rather vague, describing them as heterogeneous particles of less than 1 μm in diameter from various types of cells (Owens and Mackman, 2011; Hugel et al., 2005; Cocucci et al., 2009). Their subcellular sizes allow MPs to travel to areas where the parental cells are unable to go (e.g., extracellular spaces such as those between endothelial cells). Furthermore, the differences in their surface molecules and carried cargoes allow MPs from different lineages of parental cells to manifest diverse biological activities.

MPs shed from cell membranes are enriched in microdomains (lipid rafts), where cholesterol, phospholipids, and functional receptors are clustered (Davizon et al., 2010; Biro et al., 2005). These surface molecules often serve as signatures of the parental cells (Moskovich and Fishelson, 2007). For example, platelet MPs express the adhesion receptor glycoprotein (GP) Ib-IX-V complex, the integrin αIIbβ3, and GPVI (Horstman et al., 2004). Endothelial MPs contain e-selectin (CD62e), cadherin 5 type 2 (CD144), and endoglin (CD105) (Horstman et al., 2004; Jimenez et al., 2003). MPs arising from the lipid rafts of monocytes contain tissue factor and P-selectin glycoprotein ligand 1 (Del Conde et al., 2005). MPs can also contain genomic and mitochondrial DNA and a variety of RNA species (ribosomal, messenger, and micro) that become MP-bound during cell apoptosis (Boudreau et al., 2014; Zhao et al., 2016; Miranda et al., 2010; Cai et al., 2013; Hasselmann et al., 2001; Reich and Pisetsky, 2009).

**Figure 1:**
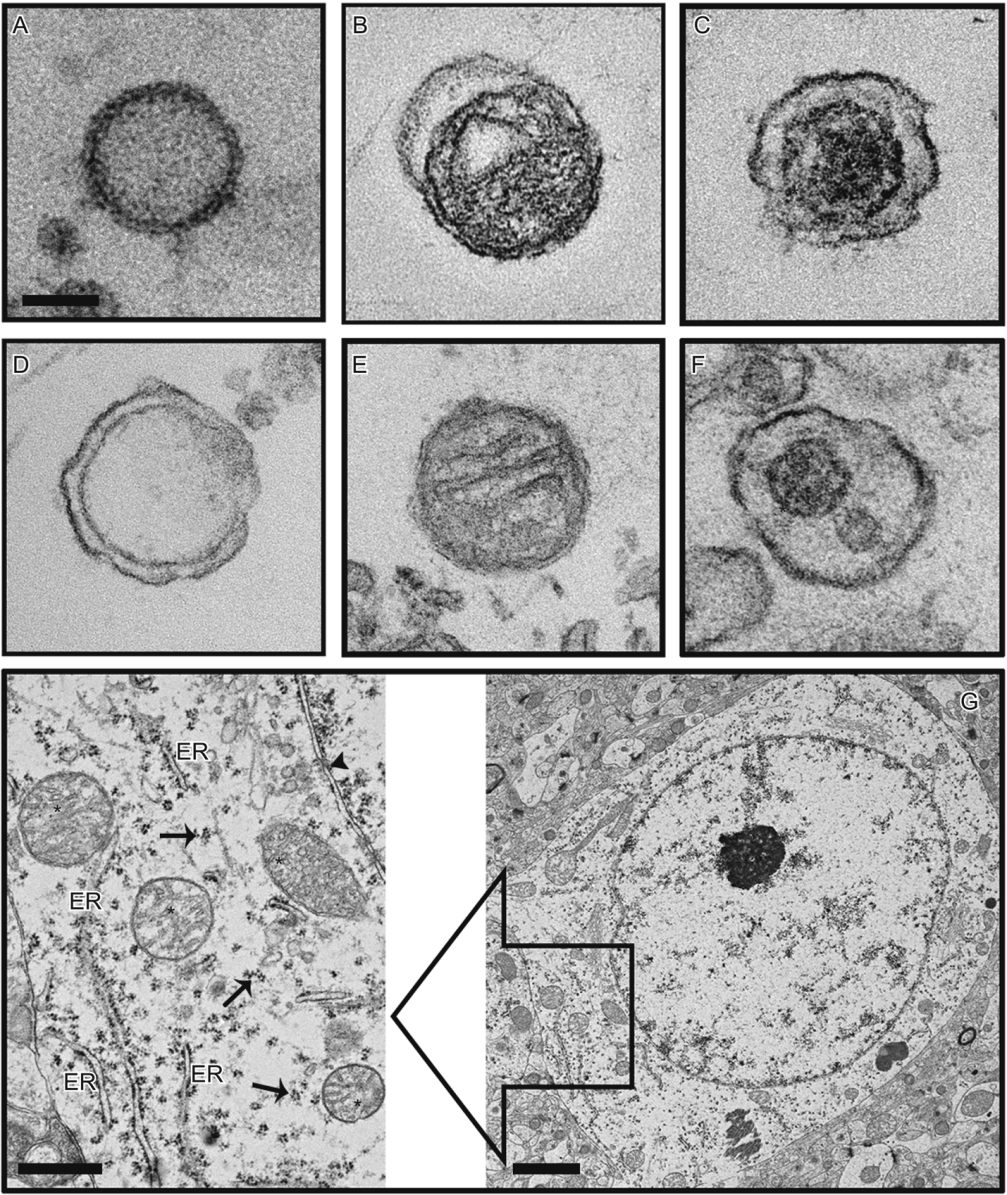
**mtMPs were detected in plasma samples from FPI mice**. TEM images of mitochondria-free MPs (A), naked mitochondria (B), and mitochondria-embedded BDMP (C*: mitochondria) detected in plasma samples of FPI mice. (D) Mitochondria-lacking MPs, mitochondria (E), and mitochondria-embedded membrane microparticles (F*: mitochondria) from BDMPs produced *in vitro*. (G) The section of a non-injured mouse brain shows a dense perinuclear accumulation of mitochondria (left, arrowhead: nuclear membrane). A locally enlarged image further shows mitochondria (*), endoplasmic reticulum (ER) and membrane-bound, and free polyribosomes (arrow)

MPs can also be intracellular granules. For example, enzyme-rich lysosomes are detected in the circulation as CD68^+^ or CD63^+^ MPs (Horstman et al., 2004). We have recently detected intact or partially damaged mitochondria as either free microparticles or those embedded in plasma membrane of parental cells in the peripheral blood samples of mice subjected to severe TBI (Fig. 1) (Zhao et al., 2016). These mitochondrial microparticles (mtMPs) account for >55% of all annexin V-binding MPs found in the peripheral blood of mice subjected to acute TBI. The membrane integrity and internal structures of mtMPs are well maintained. This predominant presence of mtMPs is consistent with neurons and glial cells being mitochondria-rich to meet the high energy needs of brain cells.

Because of their high energy consumption, brain cells are prone to the production of MPs as a result of ischemic and inflammatory insults secondary to TBI (Bianco et al., 2005; Ferrari et al., 1997; Duan and Neary, 2006). We have shown that more than 70,000/μL of brain-derived neuronal and glial cell microparticles (BDMPs) are detected in the peripheral blood of mice within hours after they are subjected to fluidic percussion injury (Tian et al., 2015). Our finding is consistent with other reports on elevated levels of MPs of various cell types in blood samples of TBI patients and animals subjected to TBI (Table 1). These BDMPs play a critical role in the development of TBI-associated coagulopathy defined as the inability of blood to clot.

**Table 1:** Microparticles as biomarkers of traumatic brain injury: studies and findings

Study	Microparticle phenotype	Findings
**Clinical studies**		
Jacoby et al. (2001)	PMP: CD61	Increased numbers of PMP in whole blood in TBI patients
Morel et al. (2008)	MP: Annexin V	Increased numbers of Annexin V^+^ MP in the CSF and in the plasma of severe TBI patients
Tschuor et al. (2008)	MP: CD61	Deceased MPs in patients’ plasma in the second week post TBI
Patz et al. (2013)	MP: miR-9, miR-451	Increased MP in CSF of severe TBI patients
Nekludov et al. (2014)	PMP: CD42a, P-selectin; EMP: CD144, TF; LMP: CD45, TF; MP: lactadherin	Increased EMP and PMP in cerebrovenous; Increased LMP in arterial in patients with severe isolated traumatic brain injury
**Animal studies**		
Midura et al. (2015)	PMP: CD41	Increased PMP in the plasma of TBI mice contributes to hypercoagulant
Tian et al. (2015)	BDMP: Annexin V, NSE, GFAP	Increased BDMP in the plasma of TBI mice
Andrews et al. (2016)	EMP: Occludin	EMP increased in plasma of TBI mice
Bohman et al. (2016)	MP: Annexin V	MP levels were elevated in the serum of TBI mice
Yasui et al. (2016)	MP: TF, GFAP	Increased TF-positive MP in the alveolar spaces of TBI rats
Zhao et al. (2016)	mtMP: MitoTracker, Annexin V, NSE,	Incresed mtMP in the plasma of TBI mice
Harrison et al. (2016)	EV: miR-212, miR-21, miR-146, miR-7a, and miR-7b	Increased miR-21, miR-146, miR-7a, and miR-7b in EV in the traumatic mice brain

**Abbreviations:** BDMP, brain-derived microparticles; CSF, cerebrospinal fluid; EMP, endothelial-derived microparticles; EV, extracellular vesicles; GFAP, glial fibrillary acidic protein; LMP, leukocyte-derived microparticles; miR, micro RNA; MP, microparticles; mtMP, mitochondrial miroparticles; PMP, platelet-derived microparticles; TBI, traumatic brain injury; TF, tissue factor; EMPs, endothelium-derived microparticles

## Microparticles and TBI-associated coagulopathy

Coagulopathy often develops in trauma patients, primarily due to substantial blood losses (hemorrhagic shock), hemodilution after substantial transfusion of crystalloids, and hypothermia (Maani et al., 2009; Wafaisade et al., 2010). It is also reported in 10% to 97.2% of TBI patients, depending on the tests used to define the coagulopathy (Harhangi et al., 2008), even though isolated TBI lacks two critical triggers of coagulopathy—a large volume of blood loss and substantial fluid resuscitation (Chang et al., 2016; Zhang et al., 2012)—suggesting that TBI-AC follows a distinct pathway that differs from coagulopathy found in trauma to the trunk and limbs (Zhang et al., 2012; Corps et al., 2015). However, despite extensive studies in the past, the pathogenesis of TBI-AC remains poorly defined.

We have recently shown that BDMPs are a major mediator of TBI-AC (Tian et al., 2015). BDMPs released into the systemic circulation induce a rapid and systemic hypercoagulable state that is quickly turned into consumptive coagulopathy, consistent with clinical observations of patients with TBI-AC (Hulka et al.,^1996^; Stein and Smith, 2004). This phenotype observed in TBI mice is reproduced in uninjured mice that have been infused with purified BDMPs, implicating an essential role of MPs in the development of coagulopathy. The procoagulant activity of BDMPs is mediated by the procoagulant anionic phospholipids, primarily phosphatidylserine (PS), that are highly enriched on the surface of BDMPs. PS is normally present on the inner leaflet (Kunzelmann-Marche et al., 2000), whereas neutral phospholipids (e.g., phosphatidylcholine) are found on the external leaflet of a membrane bilayer (Phillis et al., 2006). This asymmetry is maintained by active transporters (Devaux, 1992; Suzuki et al., 2010), but is lost in cells undergoing apoptosis or microvesiculation, leading to the exposure of PS on the outer membrane (Zwaal et al., 1977). This PS-mediated procoagulant activity is expected to be higher for BDMPs because phospholipids are highly enriched on the membrane of brain cells (Sparvero et al., 2010), accounting for ~25% of the dry weight of an adult brain compared to ~10% of other tissues (Lentz, 2003; Nesheim and Mann, 1983). We further determined that cardiolipin (CL, (sn-3’-phosphatidyl)-sn-glycerol)), which is almost exclusively located in the mitochondrial inner membrane of a normal cell (Hovius et al., 1021; de Kroon et al., 1997), is exposed on the surface of mtMPs released from traumatized brain cells (Zhao et al., 2016). It is the exposed CL that makes mtMPs procoagulant at a level comparable to that of BDMPs. Our study also suggests that the procoagulant activity of anionic phospholipids requires their proper orientation and interaction with other membrane proteins as CL exposed on mtMPs was 1,600 times as active in promoting coagulation as an equivalent number of carrier-free CL micelles (Zhao et al., 2016). The procoagulant activity of microparticles is unlikely to be limited to neuron- and glial cell-derived MPs, as PS is ubiquitously distributed on the surface of all apoptotic cells (Nagata et al., 2010). For example, PS exposed on activated platelets also makes platelet-derived MPs procoagulant (Owens and Mackman, 2011; Ding et al., 2015). These platelet-derived MPs have been detected at elevated levels in patients with TBI (Nekludov et al., 2014; Morel et al., 2008; Jacoby et al., 2001), potentially contributing to the development of TBI-AC.

In addition to their direct influence on coagulation, BDMPs and mtMPs may also bind and activate platelets as they synergize with them to disrupt the integrity of the endothelial cell junctions, thereby allowing their release into the systemic circulation (Zhao et al., 2016; Tian et al., 2015). Platelet activation has indeed been reported during the acute phase of TBI (Jacoby et al., 2001; Awasthi et al., 1991; Auer and Ott, 1979). Because of the anionic phospholipid exposure, activated platelets and platelet-derived microparticles serve as platforms to promote systemic coagulation and thrombosis (Warren and Vales, 1972). The latter is characterized by platelet-rich and fibrin-rich intravascular microthrombosis that has been reported in TBI patients (Stein and Smith, 2004; Maeda et al., 1997; Kaufman et al., 1984; Steinet al., 2004) as well as in mouse models of TBI (Zhao et al., 2016; Tian et al., 2015; Maeda et al., 1997; van der Sande et al., 1981; Stein et al., 2002; Lu et al., 2004). Consistent with this notion, platelet dysfunctions have been reported to play a causal role in TBI-AC and TBI mortality (Schnuriger et al., 2010; Nekludov et al., 2007). It is very likely that these BDMPs interact with not only platelets (Tian et al., 2015), but also ECs and other cells, to spread and exaggerate coagulation, a key feature of consumptive coagulopathy.

The detection of substantial mtMPs in the circulation of mice subjected to TBI also raises an important question regarding the oxidative modification of phospholipids and proteins. If mtMPs released into the circulation remain active in ATP production, they could produce reactive oxygen species (ROS), the byproducts of ATP production (Murphy, 2009). These redox competent mtMPs may therefore serve as a source of oxidative stress and explain why CL is a preferred oxidation substrate in the condition of TBI (Ji et al., 2012). In fact, brain phospholipids are highly susceptible to oxidative stress (Bayir et al., 2007; Tyurin et al., 2008; Huvaere et al., 2010). The lipid peroxidation markers malondialdehyde and F2-isoprostane are increased in brain tissue, serum, and CSF after TBI (Sparvero et al., 2010; Seifman et al., 2008; Hoffman et al., 1996) and are associated with poor clinical outcomes (Pilitsis et al., 2003; Kasprzak et al., 2001). Whether oxidized phospholipids are more or less active in promoting coagulation remains a subject of further investigation, but the peroxidation of membrane phospholipids has been implicated in apoptosis, mitochondrial dysfunction, and various disease states (Bochkov et al., 2010; Maki et al., 2009; Frostegard et al., 2005). Oxidative stress is also a hallmark of TBI-induced inflammation that plays a major role in the development of secondary injury to the brain and other organs after TBI (Visavadiya et al., 2016; Hiebert et al., 2015; Cavallucci et al., 2014).

## Microparticles and TBI-induced inflammation

Because of their prominent presence, redox-competent mtMPs could be a major source of oxidative stress that activates platelets (Arthur et al., 2008; Begonja et al., 2005), endothelial cells (Pearlstein et al., 2002; Li et al., 1999), neurons, glial cells (Visavadiya et al., 2016; Hiebert et al., 2015; Cavallucci et al., 2014), and the immune system (Dong, 2014) to promote inflammation. In support of this notion, multiple species of oxidized CL act as death signals for neurons as they induce the release of proapoptotic factors such as cytochrome C into the cytosol to activate caspases (Petrosillo et al., 2006; Kagan et al., 2005). This CL-induced membrane permeabilization involves several proteins on the outer membranes of mitochondria (Korytowski et al., 2011; Rostovtseva et al., 2006; Betaneli et al., 2012), consistent with our observation that CL on mtMPs is more procoagulant than purified CL micelles of equivalent concentrations. Free mitochondria released from platelets have been identified as a substrate for secreted phospholipase A2 group IIA (Boudreau et al., 2014), which is secreted by cells during the acute phase reaction (Birts et al., 2010). This phospholipase hydrolyzes the sn-2 acyl bond of glycerophospholipids to release free fatty acids and lysophospholipids to activate platelets and promote inflammation. Consistent with this proinflammatory activity, mitochondria that are intravenously injected into mice promote neutrophil activation and interaction with the endothelium (Boudreau et al., 2014).

MPs can significantly affect the immune system to propagate inflammation. MPs shed from neutrophils bind to bacteria through clusters of complement receptor 1 (Cr1), providing a robust defense against bacteria (Gasser et al., 2003; Hess et al., 1999). The Cr1 clusters are co-localized with myeloperoxidase and leukocyte elastase on the surface of MPs (Gasser et al., 2003; Hess et al., 1999), suggesting the involvement of oxidative actions during the activation of the immune system. During the acute phase reaction, neutrophil-derived MPs stimulate macrophages to release anti-inflammatory factors, such as transforming growth factor (TGF)-β, which, together with exposed PS on the surface of MPs, down-regulates inflammation (Gasser and Schifferli, 2004). By releasing anaphylatoxins upon the activation of the complement system, MPs also stimulate the host defense to initiate self-clearance through phagocytosis, thus eliminating MP-bound cytotoxin and proinflammatory mediators (Nauta et al., 2002). MPs can also carry and release the proinflammatory mediators such as IL-6, IL-1β, and CC-chemokine ligand (CCL)-2 (MCP-1) (MacKenzie et al., 2001; Mesri and Altieri, 1999), as well as vehicles to transfer chemokine receptors, such as CC-chemokine receptor 5 (CCR5) and CXC chemokine receptor 4 (CXCR4), between cells (Mack et al., 2000; Rozmyslowicz et al., 2003).

Microparticles from brain cells also have inflammation-promoting activities (Kumar and Loane, 2012; Obermeier et al., 2013). Microglia cells, which act as immune cells of the CNS, are triggered to microvesiculate when ATP binds to its receptor P2X_7_ on these cells (Bianco et al., 2005). These microglia-derived MPs contain the proprotein of the proinflammatory cytokine interleukin-1β (IL-1β), which is released upon cleavage by its processing enzyme caspase 1 (Bianco et al., 2005). A high level of microglia-derived MPs has been found in the peripheral blood and cerebrospinal fluid of patients with acute multiple sclerosis (Verderio et al., 2012; Saenz-Cuesta et al., 2014), a demyelinating autoimmune disease. Similarly, mice with autoimmune encephalomyelitis develop localized inflammation at the site where microglia-derived MPs were injected (Verderio et al., 2012). The production and biological activity of microglial MPs in the pathological course of TBI remain to be investigated. Astrocytes undergo a very similar process to generate IL-1β-containing microparticles in response to ATP (Bianco et al., 2009). The process of converting pro-IL-1β to its mature form requires the activation of acid sphingomyelinase in the outer leaflet of the plasma membrane (Bianco et al., 2009), suggesting a collaborative interaction between proteins and membrane phospholipids on the surface of MPs. Astrocytes also release mtMPs in a calcium-dependent manner that involves CD38 and cyclic ADP ribose signal (Hayakawa et al., 2016). Interestingly, these astrocyte-derived mtMPs are able to migrate into adjacent neurons to improve cell survival after ischemic stroke (Hayakawa et al., 2016). The production and activity of neuronal MPs are far less understood. It has been shown that a high level of mitochondrial Ca^2+^ increases the production of ROS and the activation of caspases, leading to neuronal apoptosis to produce MPs (Cheng et al., 2012). Neuronal MPs thus produced are reported to contain miRNA-21, which stimulates neuroinflammation and propagates damages to neurons after TBI (Harrison et al., 2016). Following membrane depolarization, cortical neurons from a mature mammalian brain release exosome-like vesicles (Lachenal et al., 2011; Faure et al., 2006) that selectively bind to adjacent neurons (Chivet et al., 2014). Exosomes isolated from the medium of primary cortical neuron cultures contain abundant microRNAs and small RNAs such as miR-124a. These exosomes can be internalized by astrocytes, leading to an increase in cytoplasm of astrocyte miR-124a and excitatory amino acid transporter 2 (EAAT2, also known as rodent analog GLT1). The latter is an important mediator of glutamate uptake in the brain (Morel et al., 2013).

**Figure 2:**
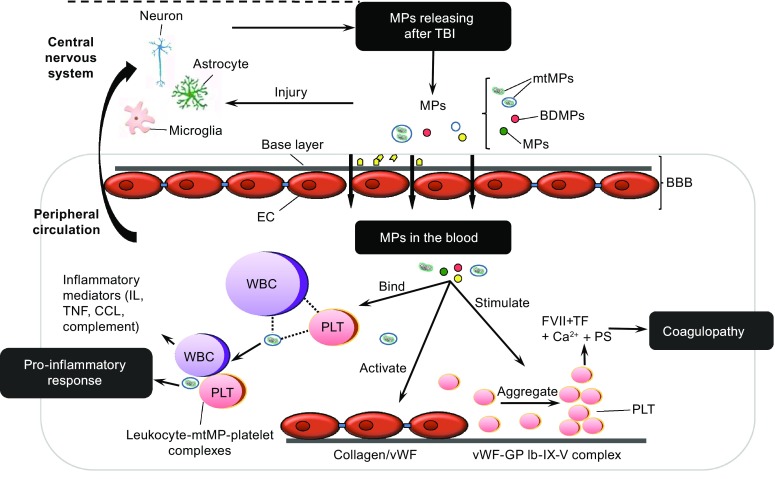
**A schematic illustration of MPs-mediated intercellular communication in TBI**. Upon injury, neurons, microglia cells, and astrocytes release MPs into peripheral blood through disrupted BBB. These BDMPs activate platelets and promote platelet adhesion to the activated endothelium and exposed subendothelium at sites of vascular injury through multiple ligand-receptor interactions. Activated platelets also provide a PS-rich surface on which tissue factor forms a complex with coagulation factor VIIa to initiate the extrinsic pathway of coagulation. MPs interact with WBCs to promote inflammation

Finally, MPs and mtMPs synergize with platelets to disrupt the blood-brain barrier (BBB) (Zhao et al., 2016; Tian et al., 2015; Pan et al., 2016), which is composed of endothelial cells, pericytes, and astrocytes and is highly susceptible to traumatic and ischemic injures (Shetty et al., 2014). Since BBB regulates communications between systemic circulation and CNS, its disruption is therefore a key event that allows BDMPs to exert systemic influences. A broken endothelial barrier will also allow peripheral molecules to affect brain functions (Kumar and Loane, 2012; Shetty et al., 2014; Shlosberg et al., 2010). This brain-body interaction is sufficiently demonstrated in the development of BDMP-induced systemic coagulopathy (Fig. 2) (Zhao et al., 2016; Tian et al., 2015) .

## Conclusion and perspectives

MPs are an emerging class of biological mediators that share phenotypic and biological characteristics of their parental cells, while also engaging in activities that are distinct from those of their parental cells. MPs mediate intercellular communication (Zhao et al., 2016; Tian et al., 2015; Budnik et al., 2016; Zappulli et al., 2016), resulting in secondary injures such as systemic coagulopathy and inflammation after TBI (Zhao et al., 2016; Tian et al., 2015). In this regard, MPs serve as functional mediators for TBI-induced injuries and their progression. They could also serve as therapeutic targets of TBI and its secondary injuries. Because of the structural complexity and multi-level activities of MPs, there are outstanding questions and challenges in applying research to clinical diagnosis and therapeutics. The foremost challenge is to identify reliable and effective means of characterizing different types of MPs and distinguishing their functional specificities. The second is to dissect communications of MPs with cells that could lead to transient or persistent phenotypic changes of targeted cells.
